# BMI and Polycystic Ovary Syndrome: Demographic Trends in Weight and Health

**DOI:** 10.7759/cureus.55439

**Published:** 2024-03-03

**Authors:** Ipsita Mohapatra, Subha R Samantaray

**Affiliations:** 1 Obstetrics and Gynecology, All India Institute of Medical Sciences, Kalyani, IND; 2 Obstetrics and Gynecology, Prathima Institute of Medical Sciences, Karimnagar, IND

**Keywords:** polycystic ovary syndrome, lean pcos, obese pcos, lifestyle modification (lsm), obesity, insulin resistance, polycystic ovary syndrome (pcos)

## Abstract

Introduction

Polycystic ovary syndrome (PCOS) is a common endocrine disorder that affects women in adolescence and reproductive age. The distribution of PCOS across different body mass index (BMI) categories can vary, and research has shown associations between PCOS and weight status. This study tries to evaluate the distribution of PCOS in relation to BMI in women attending the PCOS clinic in a tertiary hospital in eastern India.

Methodology

This hospital-based cross-sectional study was carried out in the gynecology outpatient department of a tertiary care center. The study population included all the women in the age group between 15 and 45 years diagnosed as having PCOS using the Rotterdam definition. The various physical, clinical, and biochemical parameters were measured in the study population and compared among the obese and lean PCOS patients.

Results and discussion

A total of 143 women were included in the study. The mean age of the study population was 26.8 years. Among these, the underweight and normal weight patients were categorized as lean PCOS patients, 35 in number (24.5%), and overweight and obese patients were categorized as obese PCOS patients, 108 in number (75.5%). All the physical parameter measures like age (mean = 28.05, SD = 5.722), height (mean = 153.384, SD = 6.679), weight (mean = 68.182, SD = 11.501), waist circumference (mean = 95.135, SD = 10.291), hip circumference (mean = 101.47, SD = 9.320), waist-to-hip ratio (mean = 0.940, SD = 0.0831), and neck circumference (mean = 34.85, SD = 2.445) were significantly higher in the obese group as compared to the lean group. Menstrual irregularity was significantly more common in the obese PCOS patients as compared to the lean PCOS group (p = 0.02). There was a significant difference (p < 0.05) between the obese and lean PCOS patients when the biochemical parameters like fasting insulin, fasting glucose, and homeostatic model assessment of insulin resistance (HOMA-IR) were compared. There is a strong link between obesity, insulin resistance, and PCOS. Obesity can exacerbate insulin resistance, a common feature of PCOS, leading to increased levels of insulin and androgens.

Conclusion

The demographic distribution of PCOS in relation to BMI is essential for tailoring interventions and treatments.

## Introduction

Polycystic ovary syndrome (PCOS) is a common endocrine disorder that affects women in adolescence and reproductive age. Data suggest that approximately 116 million women (3.4%) are affected by PCOS globally [1]. PCOS can have complex interactions with various factors, including weight fluctuations and lifestyle choices. The distribution of PCOS across different body mass index (BMI) categories can vary, and research has shown associations between PCOS and weight status. Obesity is highly prevalent in patients with PCOS. There is a preferential androgenic pattern of distribution of body fat in women with PCOS. These patients often present with added features of insulin resistance (IR) and hyperandrogenemia. The prevalence of obesity in the diagnosed patients of PCOS is about 30-75% [2]. PCOS is prevalent across all BMI categories but the manifestation and severity of symptoms among different BMI groups may differ. Intra-abdominal fat and obesity have a detrimental impact on tissue IR because of the increased plasma levels of non-esterified fatty acids originating from the lipolysis of abdominal fat. IR is the key factor associated with metabolic syndrome in PCOS and can lead to various cardiovascular complications and diabetes mellitus later in life. This study tries to evaluate the distribution of PCOS in relation to BMI in women attending the PCOS clinic in a tertiary hospital in eastern India. It also attempts to find the variation in clinical symptoms and biochemical parameters in different groups.

## Materials and methods

This hospital-based cross-sectional study was carried out in the outpatient department of gynecology of a tertiary care center. The study population included all the women in the age group between 15 and 45 years diagnosed as having PCOS using the Rotterdam definition [3]. According to the Rotterdam definition, two of the three criteria had to be met: oligomenorrhea or amenorrhea (less than eight menstrual cycles in 12 months or if the menstrual interval is more than 35 days), biochemical (elevated levels of total or free testosterone, androstenedione, or dehydroepiandrosterone sulfate) or clinical hyperandrogenism (such as hirsutism, male pattern of alopecia, and acne), and characteristic image of polycystic ovaries (at least one ovary containing 12 or more peripheral follicles measuring 2-9 mm in diameter and/or ovarian volume of at least 10 mL) on transvaginal or abdominal ultrasound. The transvaginal ultrasound approach was preferred in the diagnosis of PCOS, if sexually active and if acceptable to the individual being assessed. Exclusion criteria included women who had a history of diabetes mellitus, or other causes of hyperandrogenemia like congenital adrenal hyperplasia, androgen-secreting neoplasms, Cushing’s syndrome, and women aged less than 15 and more than 45 years.

An Indian study done by Kalra et al. reported that the prevalence of IR among women with PCOS is 76.9% [4]. Assuming an alpha value of 0.05 and a power of 80%, the calculated sample size was 118. Assuming a refusal rate of 20%, the calculated final sample size was 142 women with PCOS. The study was started after obtaining ethical approval from the Institute Ethics Committee of the All India Institute of Medical Sciences, Bhubaneswar. The participants were enrolled in this study after taking written informed consent. The participants were fully explained about the study procedures.

A pre-tested semi-structured questionnaire was used to collect socio-demographic details of the study participants. Obstetric index and clinical features of hyperandrogenism like acne and hirsutism were also collected by a pre-appointed nursing officer in a structured format. Anthropometric variables like height (in centimeters), weight (in kilograms), BMI, waist circumference (in centimeters), hip circumference (in centimeters), and neck circumference (in centimeters) were recorded using standard tools. BMI was calculated as weight in kilograms divided by the square of height in meters. Waist circumference was measured at the horizontal plane midway between the lowest ribs and the iliac crest. Hip circumference was measured at the level of the largest lateral extension of the hips. Neck circumference was measured in the midway of the neck, between the mid-cervical spine and mid-anterior neck, to within 1 mm, with non-stretchable plastic tape with the subjects standing upright. Biochemical parameters like fasting blood sugar and oral glucose tolerance test value with 75 grams of glucose at two hours were measured using an automated biochemical analyzer. Serum fasting insulin concentration was measured using the chemiluminescence technique. These biochemical tests were done as a part of routine investigations in PCOS patients. Ultrasonography scanning was performed in real time using a three-dimensional ultrasound machine with a high-resolution 6-MHz probe. Transvaginal ultrasonography was preferred in women who were sexually active. Assessment of the total number of ovarian follicles with a diameter between 2 and 9 mm was done using the three-dimensional sonography-based automated volume count (sono AVC) technique. Ovarian volume was estimated by marking the outlines and calculating the result using appropriate software.

The total study population was divided into four groups according to their BMI: <18.5 kg/m2, 18.5-22.9 kg/m2, 23-24.9 kg/m2, and >25 kg/m2 (underweight, normal, overweight, and obese, respectively). The underweight and normal-weight patients were categorized as lean PCOS. The overweight and obese PCOS patients were categorized as obese PCOS.

All the data were entered in Microsoft Excel 2013 (Microsoft Corporation, Redmond, WA) and analyzed using IBM SPSS Statistics for Windows, version 24.0 (IBM Corp., Armonk, NY). All the parameters of the patients were recorded as mean ± SD. The normality of quantitative data was checked by measures of Kolmogorov-Smirnov tests of normality. If data were normally distributed, an independent t-test was applied for the comparison of the two groups. Proportions were compared using the chi-square or Fisher’s exact test, whichever was applicable. All statistical tests were two-sided and performed at a significance level of p < 0.05.

## Results

A total of 143 women attending the outpatient department were diagnosed with PCOS according to the Rotterdam’s criteria. The mean age of the study population was 26.8 years, with the minimum age being 15 years; the maximum age was 42 years, with an SD of 6.102. The total study population was divided into four groups according to their BMI: <18.5 kg/m2, 18.5-22.9 kg/m2, 23-24.9 kg/m2, and >25 kg/m2 (underweight, normal, overweight, and obese, respectively). Table 1 shows the distribution of the enrolled PCOS patients into different BMI groups. Of these, underweight and normal weight patients were categorized as lean PCOS patients, 35 in number (24.5%), and overweight and obese patients were categorized as obese PCOS patients, 108 in number (75.5%).

**Table 1:** BMI distribution among the enrolled PCOS patients. BMI: body mass index; PCOS: polycystic ovary syndrome.

BMI category (kg/m^2^)	Definition	Distribution
<18.5	Underweight	0
18.5-22.9	Normal	35
23-24.9	Overweight	17
>25	Obese	88

Table 2 depicts the comparison of demographic parameters among obese and lean PCOS patients. All the anthropometric parameter measures like age, height, weight, waist circumference, hip circumference, waist-to-hip ratio, and neck circumference were significantly higher in the obese group as compared to the lean group.

**Table 2:** Comparison of the anthropometric parameters among the obese and lean PCOS patients. PCOS: polycystic ovary syndrome; WHR: waist-to-hip ratio; NC: neck circumference.

Parameters	Mean distribution (n = 143), mean (SD)	Obese PCOS (n = 108), mean (SD)	Lean PCOS (n = 35), mean (SD)	P-value
Age (years)	26.8 (6.102)	28.05 (5.722)	22.94 (5.677)	0.000
Height (cm)	154.11 (6.974)	153.384 (6.679)	156.371 (7.471)	0.027
Weight (kg)	64.215 (12.579)	68.182 (11.501)	51.974 (6.357)	0.000
Waist (cm)	89.752 (13.325)	95.135 (10.291)	73.143 (5.678)	0.000
Hip (cm)	97.56 (11.358)	101.47 (9.320)	85.49 (8.185)	0.000
WHR	0.92 (0.084)	0.940 (0.083)	0.858 (0.051)	0.000
NC (cm)	33.68 (3.446)	34.85 (2.445)	30.06 (3.594)	0.000

When we observe Table 3, we can find that menstrual irregularity is significantly more common in obese PCOS patients as compared to the lean PCOS group (p = 0.021). The presence of hirsutism, male baldness, and acne was more common in the obese PCOS group but was not statistically significant.

**Table 3:** Comparison of clinical features among obese and lean PCOS patients. PCOS: polycystic ovary syndrome.

Parameters	Category	Mean distribution (n = 143)	Obese PCOS (n = 108)	Lean PCOS (n = 35)	P-value
Menstrual irregularity	Yes	120 (83.9%)	95	25	0.021
	No	23 (16.1%)	13	10	
Hirsutism	Yes	37 (25.9%)	29	8	0.639
	No	106 (74.1%)	79	27	
Male baldness	Yes	26 (18.2%)	19	7	0.748
	No	117 (81.8%)	89	28	
Acne	Yes	45 (31.5%)	34	11	0.995
	No	98 (68.5%)	74	24	

Table 4 compares the biochemical parameters among the obese and lean PCOS groups. There was a significant difference (p < 0.05) between the obese and lean PCOS patients when the biochemical parameters like fasting insulin, fasting glucose, and homeostatic model assessment of insulin resistance (HOMA-IR) were compared. However, the study could not establish any significant difference between the levels of the two-hour oral glucose tolerance test (OGTT) in the obese and lean PCOS groups.

**Table 4:** Comparison of biochemical parameters among obese and lean PCOS patients. PCOS: polycystic ovary syndrome; OGTT: oral glucose tolerance test; HOMA-IR: homeostatic model assessment of insulin resistance.

Parameters	Mean distribution (n = 143)	Obese PCOS (n = 108)	Lean PCOS (n = 35)	P-value
Fasting insulin	13.438	14.987 ± 7.184	8.657 ± 3.167	0.000
Fasting glucose	97.908	99.766 ± 17.560	92.174 ± 15.870	0.025
2-hour OGTT	129.174	129.215 ± 14.340	129.049 ± 14.283	0.953
HOMA-IR	3.578	4.027 ± 2.299	2.193 ± 0.8972	0.000

## Discussion

PCOS can manifest during adolescence or reproductive age, and weight status during these periods may influence the severity of symptoms. Both normal-weight and overweight women can develop PCOS.

PCOS is commonly associated with overweight and obesity [5]. The prevalence of obesity among PCOS patients in our study was 75.52%, which is similar to the study done by Sachdeva et al. [6]. The risk of obesity is four times higher among patients with PCOS than among healthy controls [7]. PCOS is not limited to overweight and obese individuals. It is also diagnosed in individuals having normal weight or underweight. However, the prevalence is generally lower compared to those with higher BMI. Our study showed that out of 143 cases of PCOS; only 35 (24.47%) patients were cases of lean PCOS. The remaining 108 (75.53) patients were cases of obese PCOS. In our study, we found that obese PCOS patients have significantly higher measures of physical parameters like height, weight, waist circumference, hip circumference, waist-to-hip ratio, and neck circumference as compared to lean PCOS patients.

There is a strong link between obesity, insulin resistance, and PCOS. Excess adipose tissue can contribute to insulin resistance, which is a key factor in the development and exacerbation of PCOS symptoms [8]. HOMA-IR was used to estimate IR and the prevalence of IR in the present study was 55.94%. These results are similar to the study conducted by DeUgarte et al. [9]. There is a well-established association between PCOS and obesity. Obesity can exacerbate insulin resistance, a common feature of PCOS, leading to increased levels of insulin and androgens [10]. This can further disrupt normal ovarian function and exacerbate PCOS symptoms. Individuals with lean PCOS have a lean or normal BMI. This subgroup may experience similar hormonal imbalances but might not exhibit the same metabolic complications seen in overweight or obese individuals with PCOS. Lean individuals with PCOS may still have insulin resistance, albeit to a lesser degree than in obesity-associated PCOS. In the present study, we observed that fasting insulin, fasting glucose, and insulin resistance were significantly higher in obese PCOS patients than in lean PCOS patients.

Obese individuals with PCOS may experience more severe symptoms, including irregular menstrual cycles, hirsutism (excessive hair growth), and infertility [11]. PCOS is often associated with irregular menstrual cycles, and weight fluctuations can further impact the regularity of periods [7]. Both obesity and extreme weight loss can disrupt the hormonal balance required for regular ovulation. Obesity is linked to decreased fertility, and women with PCOS may already face challenges in this area. Weight loss in obese individuals with PCOS can positively influence fertility outcomes. In our study, we observed that menstrual irregularity was significantly higher in the obese PCOS group than in the lean PCOS group. Although the present study could not establish a significant statistical difference in other clinical parameters, it observed that the prevalence of male pattern baldness, hirsutism, and acne was higher in the obese PCOS group. Obesity can also complicate fertility issues associated with PCOS. Obesity in PCOS is often associated with additional metabolic complications, such as dyslipidemia, elevated blood pressure, and an increased risk of type 2 diabetes [12].

Managing PCOS involves a multi-faceted approach, and the strategies may vary based on the individual's BMI category. PCOS is associated with insulin resistance, hormonal imbalances, and metabolic disturbances, and interventions aim to address these underlying factors.

Weight loss, particularly through lifestyle modifications or bariatric surgery, has been shown to improve PCOS symptoms [13]. Even a modest weight loss can lead to improvements in insulin sensitivity, hormonal balance, and menstrual regularity. A diet high in refined carbohydrates and sugars can contribute to insulin resistance, which is often observed in individuals with PCOS. A diet with a lower glycemic index, rich in whole grains, fruits, vegetables, and lean proteins, may help manage insulin levels and, consequently, PCOS symptoms [14]. Sedentary lifestyles can contribute to weight gain and worsen insulin resistance [15]. Regular physical activity has been shown to improve insulin sensitivity, aid in weight management, and alleviate PCOS symptoms. Weight fluctuations and lifestyle factors play a crucial role in the manifestation and management of PCOS. Understanding these interactions is essential for developing personalized and effective approaches to address the unique needs of individuals affected by PCOS. Lifestyle modifications, including a balanced diet and regular exercise, are often key components in the holistic management of PCOS [16].

PCOS and weight fluctuations can affect body image and contribute to mental health issues [17]. Managing weight, whether through weight loss or weight gain, can have psychosocial implications that impact the overall well-being of individuals with PCOS. Both lean and obese PCOS patients should be emphasized on the importance of a balanced diet and regular exercise.

Underweight PCOS patients should focus on an adequate intake of nutrients. Sufficient calorie intake to support overall health without promoting excessive weight loss should be encouraged. Regular, moderate-intensity exercise should be done to improve insulin sensitivity and promote overall well-being. However, excessive exercise without adequate calorie intake may exacerbate hormonal imbalances [18]. Underweight individuals with PCOS may not have insulin resistance to the same extent as overweight individuals, but hormonal imbalances still need attention.

Normal-weight PCOS patients should emphasize on a moderate intake of complex carbohydrates, lean proteins, and healthy fats [19]. Excessive consumption of processed foods and sugars should be avoided. Both aerobic and resistance exercises can improve insulin sensitivity and support weight maintenance. Depending on the specific symptoms, medical interventions may be considered. These could include hormonal contraceptives for regulating menstrual cycles or medications to address specific symptoms like acne or hirsutism.

Weight loss, even a modest amount (5-10% of total body weight), can significantly improve insulin sensitivity and hormonal balance in overweight individuals with PCOS. Lifestyle modifications, including dietary changes and increased physical activity, should be encouraged. A well-balanced, low glycemic index diet should be adopted. Focus should be on whole foods, fiber-rich carbohydrates, lean proteins, and healthy fats. Monitoring portion sizes and avoiding excessive calorie intake is crucial for weight management. Regular aerobic exercise combined with strength training is beneficial. At least 150 minutes of moderate-intensity aerobic activity per week along with muscle-strengthening activities on two or more days should be done. In addition to lifestyle changes, medications such as metformin may be considered to improve insulin sensitivity [20].

Weight management is a key component in obese individuals with PCOS. Comprehensive lifestyle changes, including a calorie-controlled diet and increased physical activity, are essential [21,22]. Metformin can be added to improve the insulin sensitivity. Consideration of structured weight loss programs or interventions, supervised by healthcare professionals or nutritionists, may be beneficial. Regular monitoring of metabolic parameters, such as blood glucose and lipid levels, is important. A collaborative approach with a multidisciplinary healthcare team, including endocrinologists, dietitians, and mental health professionals can provide comprehensive care to obese PCOS patients.

The limitation of this study is the small sample size. Also, as the study is carried out at a tertiary care center, it may not be the true representative of the whole population.

## Conclusions

The demographic distribution of PCOS in relation to BMI is essential for tailoring interventions and treatments. Given the heterogeneity of PCOS, personalized care that considers individual BMI, metabolic health, and specific symptoms is crucial for effective management. In this study, we observed that though PCOS can occur in all BMI groups, it is more common in the obese BMI group. It is clear that if we target obesity, we will be able to prevent the development of IR and metabolic syndrome in PCOS patients. Additionally, further research will shed more light on the complex interplay between PCOS, BMI, and various demographic factors.
